# Case Series of Bacteremia Associated with Probiotic Use in Children after Cardiac Surgery, China

**DOI:** 10.3201/eid3201.250298

**Published:** 2026-01

**Authors:** Xiaofeng Wang, Shuo Li, Da Huo, Chenyu Li, Qian Zhang, Xu Wang

**Affiliations:** National Center for Cardiovascular Disease and Fuwai Hospital, Chinese Academy of Medical Sciences, Peking Union Medical College, Beijing, China (X. Wang, C. Li, Q. Zhang, X. Wang); Peking University First Hospital, Beijing (S. Li); Institute for Infectious Disease and Endemic Disease Control, Beijing Center for Disease Prevention and Control, Beijing (D. Huo); Capital Medical University School of Public Health, Beijing (D. Huo)

**Keywords:** probiotic bacteremia, bacteria, pediatric patients, cardiac surgery, China

## Abstract

We examined probiotic-associated bacteremia in a cohort of postoperative pediatric cardiac surgery patients in China. Among 16,436 children who underwent cardiac surgery during 2019–2024, a total of 5,034 received probiotics; 6 developed bacteremia with probiotic strains (*Bacillus subtilis*, *Bacillus licheniformis*, *Lacticaseibacillus rhamnosus*). Three cases occurred in children who had not directly received probiotics, suggesting potential cross-contamination or catheter-related transmission. All 6 patients had complex congenital heart disease and central venous catheters; 5 underwent palliative surgery. Fever, elevated C-reactive protein and leukocytes, and use of respiratory support were common. Antibiotic therapy achieved blood-culture clearance in all; 1 death occurred because of underlying cardiac disease, not infection. Our findings conclude probiotic-associated bacteremia is rare and usually resolves with antibiotics; outcomes correlate more with cardiac complexity than bacteremia itself. Maintaining perioperative probiotic use and enhancing infection-control measures, specifically regarding central line care, are recommended to minimize the risk for probiotic-associated bacteremia in pediatric cardiosurgical patients.

Probiotics are preparations containing live microorganisms that confer health benefits on the host when administered in adequate amounts (*1*). They are widely used in humans for preventing and treating various conditions, such as gastrointestinal, neonatal, allergic, and recurrent respiratory infectious diseases (*2*–*5*). However, the increasing application of probiotics has brought associated infections into focus (*6*). Some case reports indicate that the use of probiotics can lead to safety issues such as systemic infections. For instance, patients with impaired immune function can develop *Lactobacillus*-related bacteremia and endocarditis (*7*). In 2021, a systematic review analyzed 1,537 studies over a period of nearly 25 years and found 49 cases of invasive infections associated with the use of probiotics in children. Most of those infections were in children who were <2 years of age and had underlying conditions that encouraged invasive infections to develop, such as prematurity and the use of central venous catheter (CVC) (*8*).

In recent years, given progress in research on the gut microbiota of patients with congenital heart disease, the application of probiotics in patients after heart surgery has also attracted widespread attention. In a 2024 randomized controlled trial involving 112 adult congenital heart disease patients, of whom 57 were given the probiotic *Lactobacillus plantarum 24-7* and 55 were given a placebo, results showed probiotic supplementation could improve symptoms such as bloating and hard stools, and no adverse events related to probiotics were recorded (*9*). In neonatal congenital heart disease patients, a randomized controlled trial involving 100 patients, in which 50 were given the probiotic *Bifidobacterium lactis* plus inulin and 50 a placebo, the incidence of nosocomial sepsis, necrotizing enterocolitis, and death was significantly reduced in the probiotic treatment group (*10*). A randomized controlled trial involving 82 pediatric patients, 41 in the probiotics group and 41 in the placebo group, analyzed the microbiota in feces and blood, organic acid concentrations in feces, plasma intestinal fatty acid binding protein, and immunological responses (*11*). The total number of obligate anaerobes was higher in the intervention group than in the control group after postoperative day 7, and the team concluded that probiotics might alleviate intestinal damage induced by cardiopulmonary bypass in children (*11*).

As use of probiotics increases, however, cases of probiotic-associated bacteremia in children after heart surgery have been reported. For example, a 2019 study (*12*) reported the case of a 15-month-old child with dilated cardiomyopathy and severe mitral regurgitation who underwent mechanical valve replacement surgery. After surgery, the child was given probiotics to prevent antibiotic-associated diarrhea and subsequently developed fever and increased C-reactive protein (CRP) and leukocytes; blood cultures were positive for *Bifidobacterium* spp. After discontinuing probiotics and adjusting antibiotics, cultures tested negative and clinical symptoms improved (*12*). Another case was reported involving a neonate with coarctation of aorta and marginal hypoplastic left heart syndrome who underwent aortic repair and pulmonary banding operation (*13*). The neonate was given probiotics postoperatively to improve feeding intolerance and prevent necrotizing enterocolitis. The child developed increased CRP and leukocytes and thrombocytopenia; blood cultures tested positive for *Lactobacillus* (now *Lacticaseibacillus*) *rhamnosus*. Although the blood cultures became negative and acute phase reactants normalized after discontinuing probiotics and adjusting antibiotics, the child still had fever, somnolence, and hemodynamic instability and eventually died (*13*).

Such limited case reports are far from sufficient to address concerns regarding the safety of those probiotics in pediatric patients after cardiac surgery. The main obstacle to conducting clinical research is the low incidence of probiotic-associated bacteremia, which results in insufficient sample sizes to carry out high-level clinical studies. This case series study focuses on probiotic-associated bacteremia in postoperative pediatric cardiac surgery patients at a hospital in China.

## Methods

This study was conducted retrospectively and included all children who underwent cardiac surgery during 2019–2024 at the National Center for Cardiovascular Disease and Fuwai Hospital in Beijing, China. We retrieved clinical data from medical records, including demographic and surgery-related characteristics, such as age, sex, weight, cardiac malformations, surgical procedures, residual deformities after surgery (palliative surgery, arrhythmia, heart failure, pulmonary hypertension), main postoperative treatments, reasons for probiotic use, reasons for blood culture testing, respiratory and circulatory support, CRP and leukocyte levels at the time of infection, concurrent infections, antibiotic use before infection, probiotic species, possible routes of infection (probiotic use, CVC insertion), comorbidities, antibiotic therapy, and outcomes.

This hospital applies 3 different types of probiotics. The first contains *Bacillus subtilis*, *Enterococcus faecium*, and multivitamins (Beijing Hanmei Pharmaceutical Co., Ltd., https://www.bjhanmi.com.cn); the second contains *Bacillus licheniformis* and lactose (Northeast Pharmaceutical Group Shenyang First Pharmaceutical Co., Ltd., http://www.nepharm.com); and the third contains *L. rhamnosus* and *Bifidobacterium* (Nestle People’s Republic of China Co., Ltd., https://www.nestle.com). The probiotic species used in this study are closely related to strains previously reported in the literature (*1*). Although the probiotic preparations used at this hospital contain *Enterococcus faecium* and *Bifidobacterium*, those bacteria are also part of the normal gut flora. Therefore, patients with bacteremia caused by those 2 pathogens are not attributed to probiotic-associated bacteremia.

At the hospital, probiotic administration is considered in specific high-risk scenarios, such as cardiopulmonary bypass time of >120 minutes, cyanotic congenital heart disease, complex congenital heart diseases or those complicated by cardiac insufficiency, antibiotic use lasting >7 days, and the presence of intestinal dysfunction (e.g., abdominal distension and diarrhea). Conversely, probiotics are contraindicated in patients receiving immunosuppressants after organ transplantation and in those with acute intestinal diseases, such as necrotizing enterocolitis or intestinal perforation.

Catheter-related bloodstream infection was defined in accordance with the Clinical Practice Guidelines for the Diagnosis and Management of Intravascular Catheter-Related Infection: 2009 Update by the Infectious Diseases Society of America (*14*). A diagnosis requires either isolation of the same organism from >1 percutaneous blood culture and from the catheter tip (>15 CFU/mL on semiquantitative culture of a 5-cm segment) or paired blood cultures (1 from the catheter hub and 1 from a peripheral vein) that satisfy the differential-time-to-positivity criterion (e.g., microbial growth detected in the hub sample >2 hours earlier than in the peripheral sample).

This study was approved by the hospital’s ethics committee (identification no. 2024-2319). Because of the study’s retrospective nature, the requirement for informed consent was waived. The ethical principles of the 1975 Declaration of Helsinki were followed in this study.

## Results

Among 16,436 postoperative patients, 5,034 received probiotic treatment. Probiotic-associated bacteremia was documented in 6 patients, 3 of whom had not directly received probiotics. In the 3 treated patients, the causative organisms matched the administered probiotic preparations.

The ages of the 6 patients ranged from 8 months to 13 years (Table 1). Five of them received palliative surgery because of complex heart diseases. All patients had postoperative residual deformities. Three patients had a direct history of probiotic use. CVCs were inserted in all patients when the probiotic infections occurred. Fever with increased leukocyte count and CRP were the main manifestations of probiotic infection. After administration of antibiotic medications and discontinuing probiotics, 5 patients recovered (1 of whom had a cerebral embolism, manifesting in convulsions and hemiplegia), whereas 1 patient died of severe cardiac disease. In the deceased case-patient, the patient’s blood culture turned negative after a 6-day course of antibiotic therapy, indicating that the bacteremia was effectively controlled (Table 2). Nevertheless, the patient, who had a single-ventricle cardiac malformation and had undergone a Glenn procedure, subsequently developed a pulmonary embolism, which led to an increase in pulmonary vascular resistance, resulting in circulatory failure and eventual death. Therefore, the patient’s death was not directly attributable to the infection.

**Table 1:** Demographic characteristics and surgical parameters of 6 patients in study of bacteremia associated with probiotic use in children after cardiac surgery*

Category	Patient number					
	1	2	3	4	5	6
Age/sex	6 y/F	8 mo/M	8 mo/M	1.5 y/M	9 mo/F	13 y/F
Body weight, kg	21.5	5.4	5.1	11	7.2	57
Height, cm	111	63	62	85	70	150
SpO_2_, %	80	86	92	91	100	78
Cardiac disease	Single ventricle	Swiss-cheese VSD	PAA/TECD/MAPCAs	mL-TGA; TR	Dilated cardiomyopathy	DORV; complications of previous Glenn procedure; TR
Cardiac procedure	Fontan	PA-banding	RV-PA connection	DSO; tricuspid valvuloplasty	PA-banding	Tricuspid valve replacement
Palliative surgery	Yes	Yes	Yes	No	Yes	Yes
Residual deformities	Pulmonary hypertension	Increased pulmonary blood flow	Pulmonary dysplasia; MAPCAs	Cardiac dysfunction	Cardiac dysfunction	COVID-19; pulmonary embolism
Maximum VIS	29	9	14	26	8	11
Vasopressin use	Yes	No	Yes	Yes	No	Yes
Maximum CVP, mm Hg	19	4	9	13	8	12

*Patient 6 died of severe cardiac disease, not attributable to probiotic use. CVP, central venous pressure; DORV, double outlet right ventricle; DSO, double switch operation; MAPCAs, major aortopulmonary collateral arteries; mL-TGA, congenitally corrected transposition of the great arteries; PAA, pulmonary artery atresia; PA, pulmonary artery; RV, right ventricle; TECD, total endocardial cushion defect; TR, tricuspid regurgitation; VIS, vasoactive inotropic score; VSD, ventricular septal defect.

**Table 2:** Infection-related data of 6 patients in study of bacteremia associated with probiotic use in children after cardiac surgery*

Category	Patient number					
	1	2	3	4	5	6
Symptoms of onset	Fever	Fever	Fever	Fever	Fever	Fever + shock
Pathogen	*Bacillus licheniformis*	*Bacillis subtilis*	*B. subtilis*	*B. licheniformis*	*B. subtilis*	*Lacticaseibacillus rhamnosus*
Prior antibiotic exposure	Yes	Yes	Yes	Yes	Yes	Yes
Concurrent infection	No	No	*Burkholderia cepacia* (sputum)	*Acinetobacter baumanii* (blood)	No	No
Leukocyte count, × 10^9^ cells/L	16.91	27.34	13.31	41.3	5.89	26.69
Neutrophils, %	79.1	84.9	90.1	76.6	80.2	77.2
CRP, mg/L	74.9	55.1	23.8	99.9	155	305
CRBSI	No	No	No	No	Yes	No
Probiotic use	No	Yes	No	Yes	No	Yes
Probiotic duration, d	No	6	No	10	No	6
Abdominal distension/diarrhea	No	Yes	No	Yes	No	Yes
Respiratory support during infection	Mechanical ventilation	High-flow nasal cannula	Mechanical ventilation	Mechanical ventilation	No	Mechanical ventilation
VIS during infection	14	No	6	10	No	4
CVC insertion	Yes	Yes	Yes	Yes	Yes	Yes
Antibiotic	Imipenem/ cilastatin; vancomycin	Linezolid; cefoperazone/ sulbactam	Meropenem	Meropenem vancomycin	Piperacillin/ tazobactam	Meropenem
Antibiotic duration, d	10	12	10	10	7	12
Outcomes	Discharged at 65 dpo	Cerebral embolism, discharged at 136 dpo	Discharged at 71 dpo	Discharged at 153 dpo	Discharged at 42 dpo	Died 12 d after infection

*CRBSI, catheter-related bloodstream infection; CRP, C-reactive protein; CVC, central venous catheter; dpo, days postoperative; VIS, vasoactive inotropic score.

## Discussion

In this study, we investigated probiotic-associated bacteremia in children after cardiac surgery. The incidence we observed is similar to the result of a recent literature report (5 blood culture–positive *Clostridium butyricum* bacteremia cases from a total of 6,576 persons who had blood cultures positive for any bacteria) (*15*). Probiotic-associated bacteremia in pediatrics was mainly reported in premature infants, most likely attributable to severe underlying diseases and compromised immune function (*16*). Patients undergoing cardiac surgery are susceptible to a dysregulated inflammatory response because of surgical stress. That response can lead to systemic complications such as immunosuppression and impaired intestinal epithelial barrier function, thereby increasing the risk for various infections, including probiotic-associated bacteremia (*17*).

Of note, probiotics possess distinct characteristics that differentiate them from common pathogenic microorganisms. The highly acidic environment of human gastric juice is lethal to most common pathogens. Probiotics, however, possess substantial acid tolerance, enabling them to survive such bactericidal activity, reach the intestines intact, and subsequently colonize to exert their beneficial effects (*18*,*19*). If reduced immune function combined with impaired intestinal epithelial barrier function occurs in children after cardiac surgery, enterogenous bacteremia can occur.

In this study, 5 of the 6 children underwent palliative surgeries because of complex cardiac diseases. Their cardiopulmonary functions were not corrected after the operations. They still had many problems, such as residual hypoxemia, heart failure, and pulmonary hypertension (patients nos. 1, 3, and 6 received vasopressin treatment). Another child (patient no. 4), although having undergone corrective surgery, had left ventricular dysfunction because of preoperative atrioventricular valve regurgitation. The child also required a long period of extracorporeal membrane oxygenation support after the surgery. This constellation of severe, unresolved cardiopulmonary impairments provided a plausible pathological foundation for probiotic-associated bacteremia. The presence of those high-risk factors collectively pointed to bacterial translocation as a key underlying mechanism. Consequently, their infections are more likely attributable to their critical underlying conditions than to a direct pathogenic effect of the probiotics.

Moreover, those patients often require the insertion of CVCs postoperatively, which further increases the risk for infections (*20*). Patient no. 5 serves as a pertinent example of this scenario, because the infection occurred without documented probiotic use and yet in the absence of other recognized translocation risks. In this patient, after the isolation of *B. subtilis* from a percutaneous blood culture, the same organism was isolated from the catheter tip, with semiquantitative culture yielding >15 CFUs. Those findings confirmed a diagnosis of catheter-related bloodstream infection. Similar routes of transmission have been reported in previous literature, but without discussion on infection control recommendations. This case prompted a detailed investigation into potential transmission routes. After detailed investigation and analysis, we found that, in probiotic preparations, some spore-forming organisms are not sensitive to common disinfectants (such as alcohol); the alcohol-based hand rub is less reliable than soap-and-water hand hygiene and sporicidal environmental cleaning (*21*). Those precautions, however, should not be generalized to non–spore-forming probiotics, because *Clostridioides difficile*–like precautions are intended specifically for cases of suspected spore transmission.

In 2 specific cases, the patients had no history of probiotic intake and no positive CVC cultures, so the route of transmission remained undetermined. We hypothesized that this missing information could be related to the preparation of probiotic medications, which at the time were not handled separately from other oral drugs in the hospital, creating a risk for cross-contamination. This notion was supported by the occasional detection of *B. subtilis* in routine environmental surveillance.

Because this hospital does not routinely perform antimicrobial susceptibility testing on probiotic strains, such data are unavailable to guide therapy. In clinical practice, therapeutic strategy is based on the identified pathogens from blood culture, supplemented by a review of the relevant literature (*22*–*24*). This process allows for the empirical selection of appropriate antibacterial agents to eliminate the bacteremia. Treatment efficacy is subsequently evaluated through clinical indicators, such as body temperature, leukocyte, CRP, and follow-up blood culture results. Although this process constitutes an empirical approach, the resolution of bacteremia (as confirmed by negative blood cultures in all patients) demonstrates its clinical acceptability. Given the rarity of probiotic-associated bacteremia and its successful resolution with antibiotic therapy without direct adverse outcomes, we do not recommend altering the current use of probiotics in post–cardiac surgery patients.

The first limitation of this study is that, because a means of genetic strain testing (e.g., whole-genome sequencing) was not available, we cannot definitively confirm that the bacteremia originated from the administered probiotic preparations. Second, because of the small number of cases, we were unable to adequately control for potential confounders such as disease severity, immunosuppression, central venous catheter use, prolonged intensive care unit stay, or prior antibiotic exposure, which might have influenced the development of probiotic-associated bacteremia. Therefore, the findings can only suggest an association rather than establish causality. Finally, in 2 patients, other pathogens (*Burkholderia cepacia* and *Acinetobacter baumanii*) were concurrently isolated from blood or sputum, making it impossible to determine which bacterium was responsible for clinical deterioration.

In conclusion, this single-center case series found probiotic-associated bacteremia to be a rare occurrence. Among the identified cases, clinical outcomes were more closely linked to patients’ underlying complex cardiac conditions than to the bacteremia itself. On the basis of those findings, probiotics appear to be generally safe for use in pediatric patients undergoing cardiac surgery. In addition to the gastrointestinal tract, CVCs might also serve as potential routes of transmission. Therefore, enhanced infection prevention and control measures, specifically regarding central line care, are warranted in pediatric cardiosurgical patients to minimize the risk for probiotic-associated bacteremia.
